# Malignant Fibrous Histiocytoma, Aggressive Fibromatosis and Benign Fibrous Tumors Express mRNA for the Metalloproteinase Inducer *EMMPRIN* and the Metalloproteinases *MMP-2* and *MT1-MMP*

**DOI:** 10.1080/13577140120048601

**Published:** 2001-09

**Authors:** Jan Åhlén, Ulla Enberg, Catharina Larsson, Olle Larsson, Tony Frisk, Otte Brosjö, Anette von Rosen, Martin Bäckdahl

**Affiliations:** 1 Department of Surgery Karolinska Hospital Stockholm SE- 171 76 Sweden; 2 Department of Molecular Medicine Karolinska Hospital Stockholm SE- 171 76 Sweden; 3 Department of Oncology and Pathology Karolinska Hospital Stockholm SE- 171 76 Sweden; 4 Oncology Service Department of Ortopedics Karolinska Hospital Stockholm SE- 171 76 Sweden

## Abstract

*Purpose:* Extracellular matrix metalloproteinase inducer (EMMPRIN) has been shown to stimulate fibroblasts to production of matrix metalloproteinases (MMPs). MMPs comprise a family of proteolytic enzymes implicated in the degradation of extracellular matrix which has been proposed to be one of the essential steps in tumor invasion and metastases. In the present study we investigated the expression and location of mRNAs for *EMMPRIN*, matrix metalloproteinase-2 (*MMP-2*), and membrane-type 1 matrix metalloproteinase (*MT1-MMP*) in mesenchymal tumors with different tendencies to recur or metastasize.

*Subjects:* Eight malignant fibrous histiocytomas (MFH), seven aggressive fibromatosis (AF), and six benign fibrous tumors (BF).

*Method:* The mRNA-expression of *EMMPRIN*, *MMP-2* and *MT1-MMP* were studied using mRNA *in situ* hybridization technique.

*Results:* The mRNA-expression of *EMMPRIN*, *MMP-2* and *MT1-MMP* respectively were found at varying frequency and level in all tumor types. The mRNAs corresponding to *EMMPRIN* and *MMP-2* were seen in neoplastic cells as well as in endothelial cells both inside and outside the tumor pseudo-capsule, whereas *MT1-MMP* was seen only within the tumors. The estimated mRNA levels of *EMMPRIN* and *MMP-2* covariated significantly. Overall, the highest expression was found in the MFH tumors and the lowest levels in the BF tumors.

*Discussion:* These findings suggest that the MMP-inducer *EMMPRIN* and the extracellular matrix degrading system involving the metalloproteinases *MMP-2* and *MT1-MMP* is frequently activated in mesenchymal tumors. The covariation between *EMMPRIN* and *MMP-2* support previous findings that EMMPRIN may be an inducer of MMP-2. The high levels of *MMP-2* mRNA in MFH indicate a relationship between the proteolytic activity of *MMP-2* and the tumor aggressiveness.


*Sarcoma* (2001) 5, 143–149

ORIGINAL ARTICLE

### Malignant fibrous histiocytoma, aggressive fibromatosis and benign fibrous tumors express mRNA for the metalloproteinase inducer *EMMPRIN* and the metalloproteinases *MMP-2 and MT1-MMP*

JAN ÅHLÉN[1], ULLA ENBERG[1], CATHARINA LARSSON[2], OLLE LARSSON [3], TONY FRISK[2], OTTE BROSJÖ[4], ANETTE VON ROSEN[1] & MARTIN BÄCKDAHL[1]

[1]*Department of Surgery, Karolinska Hospital, SE- 171 76, Stockholm, Sweden,* [2]*Department of Molecular Medicine, Karolinska Hospital, SE- 171 76, Stockholm, Sweden,* [3]*Department of Oncology and Pathology, Karolinska Hospital, SE- 171 76, Stockholm, Sweden,* [4]*Oncology Service, Department of Ortopedics, Karolinska Hospital, SE- 171 76, Stockholm, Sweden*

**Abstract**

*Purpose:* Extracellular matrix metalloproteinase inducer (EMMPRIN) has been shown to stimulate fibroblasts to production of matrix metalloproteinases (MMPs). MMPs comprise a family of proteolytic enzymes implicated in the degradation of extracellular matrix which has been proposed to be one of the essential steps in tumor invasion and metastases. In the present study we investigated the expression and location of mRNAs for *EMMPRIN*, matrix metalloproteinase-2 (*MMP-2*), and membrane-type 1 matrix metalloproteinase (*MT1-MMP*) in mesenchymal tumors with different tendencies to recur or metastasize.

*Subjects:* Eight malignant fibrous histiocytomas (MFH), seven aggressive fibromatosis (AF), and six benign fibrous tumors (BF).

*Method:* The mRNA-expression of *EMMPRIN*, *MMP-2* and *MT1-MMP* were studied using mRNA *in situ* hybridization technique.

*Results:* The mRNA-expression of *EMMPRIN*, *MMP-2* and *MT1-MMP* respectively were found at varying frequency and level in all tumor types. The mRNAs corresponding to *EMMPRIN* and *MMP-2* were seen in neoplastic cells as well as in endothelial cells both inside and outside the tumor pseudo-capsule, whereas *MT1-MMP* was seen only within the tumors. The estimated mRNA levels of *EMMPRIN* and *MMP-2* covariated significantly. Overall, the highest expression was found in the MFH tumors and the lowest levels in the BF tumors.

*Discussion:* These findings suggest that the MMP-inducer *EMMPRIN* and the extracellular matrix degrading system involving the metalloproteinases *MMP-2* and *MT1-MMP* is frequently activated in mesenchymal tumors. The covariation between *EMMPRIN* and *MMP-2* support previous findings that EMMPRIN may be an inducer of MMP-2. The high levels of *MMP-2* mRNA in MFH indicate a relationship between the proteolytic activity of *MMP-2* and the tumor aggressiveness.

### Introduction

Soft tissue sarcomas constitute a heterogeneous and complex group of malignant tumors of mesenchymal origin which can potentially develop anywhere in the human body. A multitude of entities are described, the recognition of which is essential for their proper treatment and clinical handling. Today, surgery is the mainstay treatment for all mesenchymal tumors and there are no other generally applied effective treatments. Although surgery is often extensive, there is a need for better complements to the surgical treatment, and hence a demand for the development of reliable prognostic markers in the planning of treatment.

The most common type of soft tissue sarcoma is malignant fibrous histiocytoma (MFH), which accounts for 25–40% of all cases in adulthood.[1,2] This entity is generally characterized by aggressive biological behaviour, and the patients frequently develop distant metastases and local recurrence. The majority of cases are of high malignancy grade (grade 3 or 4 on a four-grade scale,[3–4]) and the reported 5-year survival rates vary between 50% and 70%.[5–7] Today the most important prognostic factors include tumor size, localization and histopathological grade.[1–4] The value of molecular assays has become evident to improve diagnostic sensitivity and specificity, and to achieve an improved understanding of the molecular mechanisms involved in the tumor

Address for correspondence and requests for reprints: Jan Åhlen MD Department of Surgery; Karolinska Hospital; SE-171 76 Stockholm; Sweden Tel: + 46 8 51772477; Fax: + 46 8 331587; E-mail: jan.ahlen@kirurgi.ki.se

1357–714X print/1369–1643 online/01/030143-07 © 2001 Taylor & Francis Ltd
DOI: 10.1080/13577140120071687

development and progression. Aggressive fibromatosis (AF) is a fibrous tissue tumor that usually occurs in the subfascial tissue of the abdominal wall, shoulder or chest wall. It never metastasizes but local recurrence is often seen after surgical excision. Benign fibrous tumors (BF) usually develop in the subcutaneous tissue or tendon sheath but rarely recur and never metastasize.

Interactions between the tumor cells and the stroma leading to degradation of the basement membrane and the stromal extracellular matrix play important roles in the metastatic process of many types of tumors. Matrix metalloproteinase-2 (MMP-2, 72kDa type IV collagenase, Gelatinase A) and membrane-type 1 matrix metalloproteinase (MT1-MMP), are members of a family of zinc-dependent proteolytic enzymes that degrade extracellular matrix proteins, glycoproteins and proteoglycans and are implicated in the extracellular matrix (ECM) remodelling and degradation processes.[8,9] MMP-2 degrades type IV collagen which is unique to the basement membrane, and has thus been shown to be of importance for tumor cell invasion and metastases.[10,11] It is secreted as a latent pro-enzyme which is activated on the cell surface by a complex consisting of MT1-MMP, a metalloproteinase with a transmembrane domain,[12] and tissue inhibitors of metalloproteinase type 2 (TIMP-2), a natural inhibitor of MMPs.[13] *EMMPRIN* is a transmembrane glycoprotein belonging to the superfamily of immunoglobulins. It is attached to the surface of many types of malignant human tumor cells.[14,15] *EMMPRIN* has been shown to stimulate fibroblasts to produce MMPs and is thus proposed to regulate the MMP production during tumor invasion.[16–18]

The purpose of this study was to investigate the involvement of these genes by measuring the mRNA expression of *EMMPRIN*, *MMP-2*, and *MT1-MMP* in mesenchymal tumors with different clinical outcome and with different tendency to recur or metastasize.

### Patients and tumor material

The 21 patients were all operated on at the Karolinska Hospital, and none of the patients had received any postoperative treatment. Patient and tumor data are given in Table 1. All tumors were re-evaluated and classified, according to established histopathological criteria,[1] by an experienced histopathologist who had no knowledge of the clinical course. The malignancy grading was determined on a four-grade scale based on the estimation of cellularity, cellular atypia, necrosis and mitotic frequency.[3,4] The surgical margins were also re-evaluated. The study included eight cases with MFH (four storiform-pleomorphic, two myxoid and two giant cell type), seven cases of aggressive fibromatosis (AF), and six benign fibrous tumors (BF) (two fibrous histiocytomas and four fibromas of the tendon sheath). All MFH patients were operated on in 1990, and those with AF or BF were operated on between 1990 and 1995.

### Methods

### *RNA probe preparation*

For RNA preparation of antisense and sense probes full length cDNA of *EMMPRIN* (1.6 kb) and *MMP-2* (1.2 kb) were subcloned into bluescript transcription vectors. The constructs were linearized with the proper restriction enzymes for RNA probe transcription. An *MT1-MMP* cDNA fragment (nt 1647–2889) was subcloned into the pGEM 4 vector, and antisense (405bp) and sense (837 bp) probes were transcribed. The transcriptional products were designed to eliminate the risk of cross-reactivity. The cDNA plasmids were generously supplied by Huiming Guo, Tufts University, Boston, Massachusetts, USA (*EMMPRIN*),[16] Gregory I. Goldberg, Washington University, St. Louis, MO, USA (*MMP-2*)[19] and Hiroshi Sato, Kanazawa University, Ishikawa, Japan (*MT1-MMP*).[12] The sense RNA probes were used as internal negative controls for each hybridization reaction. Hybridization to adrenocortical cancer tissue was used as a positive control for the *EMMPRIN* probe, and to breast carcinomas for *MMP-2* and *MT1-MMP*. In addition hybridization with a *β-actin* probe was used as a positive control of RNA presence in all tumors. *In vitro* transcribed RNA was labelled with $^{35}$S-UTP (10mCi/ml) using the conditions and reagents recommended by the manufacturer (Promega, Madison WI, USA), and purified by ultrafiltration (Microcon 100, Amicon, Beverly, MA, USA) prior to the hybridization reactions.

### *RNA* in situ *hybridization*

The hybridization procedures used in this study were essentially as previously described.[20] Paraffin-embedded sections of 5 μm were deparaffinized with limonene, rehydrated through graded ethanol followed by phosphate buffered saline at room temperature (RT), treated with proteinase-K (1 μg/ml, 30 minutes, 37°C), and then treated for 10 minutes with 0.1 M triethanolamine buffer (pH 8.0) containing 0.25% acetic anhydride to reduce background. The sections were then washed twice in 2× SSC, dehydrated in graded ethanol, air dried, and heated to 60°C for 30 minutes. The hybridization solution (HS) containing 50% formamide, 2× SSC, 20mM Tris-HCl pH 8.0, 1× Denhardt's solution, 1 mM EDTA, 10% dextran sulfate, yeast tRNA 500μg/ml mixed with 100mM dithiothreitol (DDT), and 2.5 × $10^{3-4}$ cpm/μl $^{35}$S-labeled RNA probe was preheated to 68°C for 10 minutes and cooled down to RT on ice. The tumor sections were covered with HS, and incubated at 55°C overnight in a humidified chamber. After hybridization, stringent washing was performed with SSC and 10mM DDT, the most stringent step being 0.1× SSC for 15 minutes at 60°C. Non-specific binding was reduced by incubating the sections in RNAse buffer (0.5 M NaCl, 10 mM Tris-HCl pH 8.0, 1 mM EDTA) and RNAse A (20 μg/ml) for 30 minutes at 37°C followed by washes with RNAse buffer and SSC in decreasing concentrations, the last two steps consisting of 0.1× SSC for 15 minutes at 60°C and for 30 minutes at RT. The sections were then dehydrated in ethanol, air dried, dipped in Kodak NTB-2 emulsion, exposed for 5–12 days *(MMP-2)*, 5–13 days *(EMMPRIN)*, and 6–38 days *(MT1-MMP)* respectively, developed, and counter-stained with hematoxylin-eosin. The sections were evaluated with both light and darkfield microscopy. Evaluation of the hybridization results were performed by three investigators including a pathologist who also re-evaluated the histopathological diagnosis. In the evaluation of *MT1-MMP*, five of the 21 tumors had positive signals for the sense probe and were therefore excluded. The expression of mRNA was graded as – = no expression above background; + = low, low expression in few cells (<10%); ++ = moderate, moderate to high expression in many cells (10–70%); and +++ = high, high expression in the majority of cells or in all cells (>70%) (Table 2–4). The classification was based on the areas with the most pronounced expression and examples of the scoring are illustrated in Figure 1.

### *Statistical analyses*

The relationship between high mRNA-expression of *EMMPRIN*, *MMP-2* or *MT1-MMP* and tumor type was analysed using the Fisher's exact test. The association between high mRNA levels and clinical outcome in MFH patients was analysed using Kaplan-Meier log-rank survival curves and Log-Rank test to evaluate the difference between the curves. The correlation between *EMMPRIN* and *MMP-2* was analysed using Spearman Rank order. All analyses where made in STATISTICA 5.5 software. Probabilities of <0.05 were accepted as statistically significant.

### Results

The mRNA-expression of *EMMPRIN*, *MMP-2* and *MT1-MMP* were analysed in twenty-one mesenchymal tumors by mRNA *in situ* hybridization technique. The clinical data, outcome and histopathology are given for each case in Table 1. Three of the eight patients with MFH died of the disease during follow-up. One patient had a local recurrence which was treated surgically, he has thereafter no evidence of disease at 6 years follow-up (Case no. 4, Table 1). The remaining four patients with MFH were without any evidence of the disease after a mean follow up of 7.5 years. The mean follow-up for the seven AF patients was 4 years during which one of the tumors recurred locally (Case no. 13, Table 1). None of the six BF recurred (Table 1).

**Table 1.** *Clinical data for the 21 mesenchymal tumors in the study*

| Case no. | Age/ sex | Diagnosis | Histologic Subtype | Grade (1–4) | Localisation | Surgical margin | Metastases | Local rec. | Outcome | Follow up (months) |
|---|---|---|---|---|---|---|---|---|---|---|
| 1 | 40/M | MFH | SP | 3 | Lower extr | Intralesional | yes | no | DOD | 9 |
| 2 | 74/F | MFH | SP | 3 | Lower extr | Wide | yes | no | DOD | 61 |
| 3 | 69/F | MFH | myxoid | 3 | Upper extr | Wide | yes | no | DOD | 57 |
| 4 | 67/M | MFH | GC | 4 | Upper extr | Wide | no | yes* | NED | 75 |
| 5 | 76/M | MFH | SP | 4 | Lower extr | Wide | no | no | NED | 81 |
| 6 | 69/F | MFH | SP | 3 | Shoulder | Wide | no | no | NED | 89 |
| 7 | 51/M | MFH | myxoid | 1 | Lower extr | Wide | no | no | NED | 91 |
| 8 | 25/F | MFH | GC | 3 | Upper extr | Marginal | no | no | NED | 98 |
| 9 | 35/F | AF | | – | Trunk wall | Marginal | no | no | NED | 60 |
| 10 | 26/M | AF | | – | Shoulder | Marginal | no | no | NED | 36 |
| 11 | 49/F | AF | | – | Shoulder | Intralesional | no | no | NED | 36 |
| 12 | 46/F | AF | | – | Lower extr. | Marginal | no | no | NED | 12 |
| 13 | 40/M | AF | | – | Shoulder | Marginal | no | yes | NED | 96 |
| 14 | 33/F | AF | | – | Thoracic wall | Intralesional | no | no | NED | 60 |
| 15 | 52/F | AF | | – | Shoulder | Wide | no | no | NED | 60 |
| 16 | 63/M | BF | FT | – | Knee | Marginal | no | no | NED | 96 |
| 17 | 65/F | BF | FH | – | Neck | Marginal | no | no | NED | 60 |
| 18 | 39/M | BF | FT | – | Hand | Marginal | no | no | NED | 84 |
| 19 | 27/M | BF | FT | – | Thumb | Marginal | no | no | NED | 120 |
| 20 | 44/F | BF | FT | – | Finger | Marginal | no | no | NED | 96 |
| 21 | 28/F | BF | FH | – | Thigh | Marginal | no | no | NED | 96 |

*Local recurrence after 11 months, thereafter no signs if diseases. MFH = malignant fibrous histiocytoma; AF = aggressive fibromatosis; BF = benign fibrous tumor; SP = storiform-pleomorphic; GC = giant cell type; FH = fibrous histiocytoma; FT = fibroma of tendon sheath; NED = no evidence of disease; DOD = dead of disease.

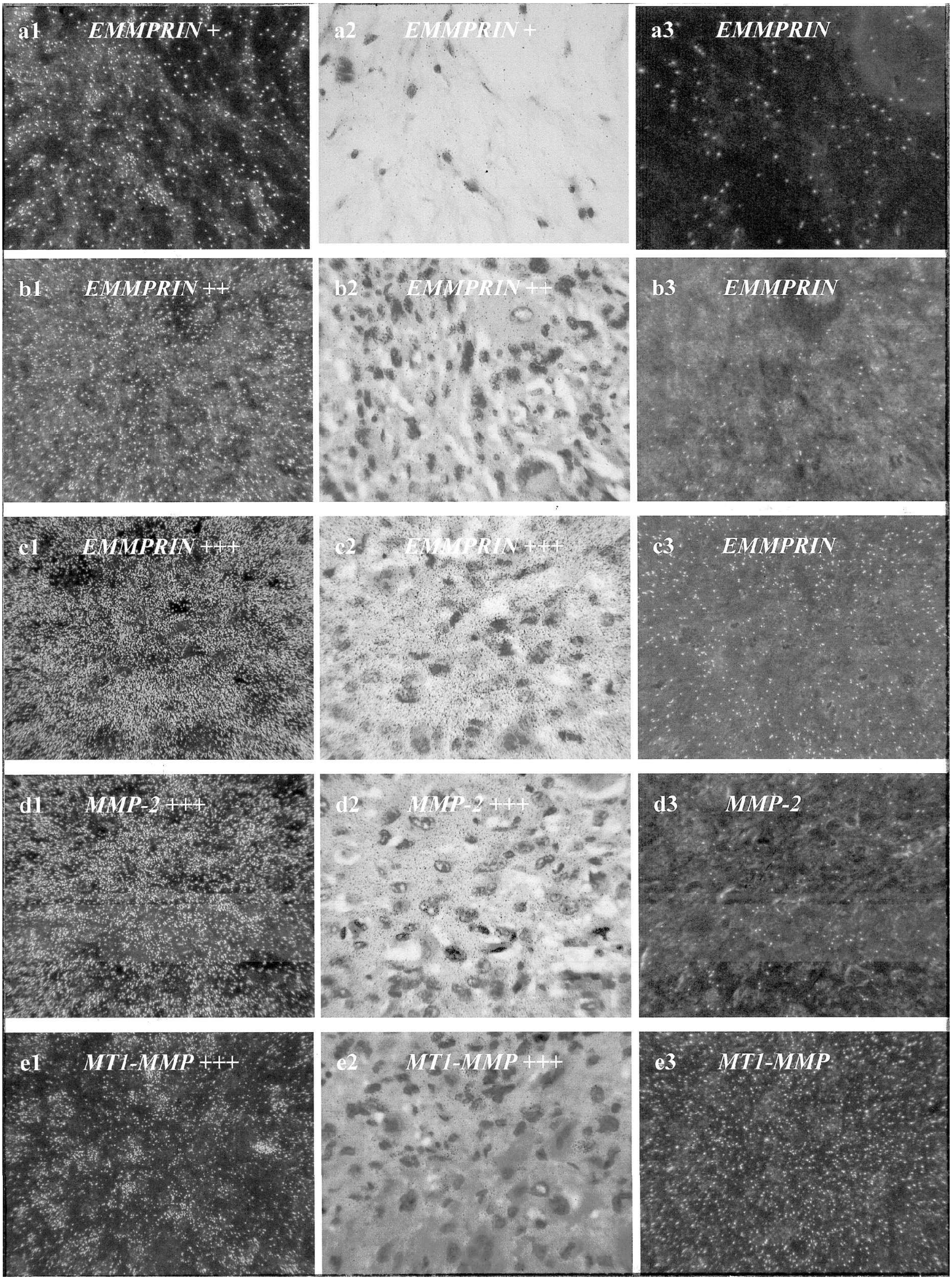

**Fig. 1.** *mRNA in situ hybrization analyses showing expression of EMMPRIN in case no 7 (a1, a2) and case no 5 (b1, b2), and of EMMPRIN, MMP-2, MT1-MMP in case no 4 (c1, c2, d1, d2, e1, e2). Hybridization with sense probe were used as negative controls (a3–e3). The hybridization signals appear as white spots in dark field microscopy (a1–e1, a3–e3) and as black spots in light field microscopy (a2–e2). The scoring of the expression is according to Table 2, and the magnification was ×200.*

The results from the mRNA *in situ* hybridization are detailed in Table 2. *β-actin* was used as positive control and was expressed in all 21 tumors analysed. Expression of both *EMMPRIN*, *MMP-2*, and *MT1-MMP*-mRNA, were found at varying frequencies and levels in all tumor types. Expression was most

**Table 2.** *Results from the mRNA in situ hybridisation analyses*

| Case no. | Type | Outcome | mRNA expression of *EMMPRIN* | *MMP-2* | *MT1-MMP* |
|---|---|---|---|---|---|
| 1 | MFH | DOD | +++ | +++ | PS |
| 2 | MFH | DOD | +++ | +++ | ++ |
| 3 | MFH | DOD | +++ | +++ | PS |
| 4 | MFH | NED* | +++ | +++ | +++ |
| 5 | MFH | NED | ++ | +++ | ++ |
| 6 | MFH | NED | ++ | +++ | + |
| 7 | MFH | NED | + | ++ | + |
| 8 | MFH | NED | - | ++ | PS |
| 9 | AF | NED | +++ | +++ | + |
| 10 | AF | NED | +++ | +++ | + |
| 11 | AF | NED | +++ | +++ | + |
| 12 | AF | NED | ++ | +++ | - |
| 13 | AF | NED* | - | + | + |
| 14 | AF | NED | + | + | PS |
| 15 | AF | NED | + | - | - |
| 16 | BF | NED | +++ | ++ | - |
| 17 | BF | NED | ++ | + | PS |
| 18 | BF | NED | ++ | ++ | ++ |
| 19 | BF | NED | - | - | ++ |
| 20 | BF | NED | - | - | - |
| 21 | BF | NED | - | - | - |

MFH = malignant fibrous histiocytoma; AF = aggressive fibromatosis; BF = benign fibroma; DOD = dead of disease; NED = no evidence of disease; – = no expression above background; + = expression in few cells, <10%; ++ = expression in many cells, 10–70%; +++ = expression in the majority of cells, >70%; PS = positive sense
*Case no. 4 and 13 have had local recurrence commonly seen in the MFH tumors, where *EMMPRIN* mRNA was expressed in seven of the eight cases. *MMP-2* mRNA was expressed in all eight cases and *MT1-MMP* mRNA was expressed in all six tumors that not had positive sense. The levels of expression were also comparatively high in the MFH tumors, with high expression (+++) detected for *EMMPRIN* mRNA in 4/8 cases and for *MMP-2* mRNA in 6/8 cases. No correlation could be seen between expression levels and the different histo-pathological subtypes. In the AF tumors *EMMPRIN* and *MMP-2* were detected in six of the seven cases, and in three and four cases respectively the detected level of expression was high (+++). *MT1-MMP* was only expressed at low levels (+) in AF. Overall the lowest levels of expression were seen in BF where expression of *EMMPRIN* and *MMP-2* were seen in three and of *MT1-MMP* in two of the six tumors.

For all three types of tumors the mRNA expression of *EMMPRIN* and *MMP-2* showed a significant tendency to covariate (correlation coefficient = 0.805, $p<0.001$). This was illustrated by e.g. the high expression (+++) in MFH cases 1–4 and AF cases 9–11 in which both genes were expressed at high levels (+++). Similarly BF tumors no 16–18 all expressed *EMMPRIN* and *MMP-2* mRNA, while the other three BF cases were negative for both genes.

*EMMPRIN*-, *MMP-2*- and *MT1-MMP* mRNAs were all identified in neoplastic cells. *EMMPRIN*- and *MMP-2* mRNAs but not *MT1-MMP* mRNA were also seen in endothelial cells. The level of expression in endothelial cells was almost identical to the overall expression. In some of the MFH tumors, an intratumoral variation of expression was found. Where normal tissue could be identified outside the tumor pseudo-capsule, *EMMPRIN*- and *MMP-2* mRNA were commonly expressed in stroma-like cells as well as in endothelial cells. Unlike *EMMPRIN* and *MMP-2*, expression of *MT1-MMP* mRNA was never seen outside the pseudo-capsule. No mRNA-expression of *EMMPRIN*, *MMP-2* or *MT1-MMP* was detected in muscle cells or in adipose tissue outside the tumor pseudo-capsule.

When high tumor mRNA-expression of *EMMPRIN*, *MMP-2* or *MT1-MMP* were compared with clinical outcome and tumor type some statistically significant associations were revealed. High levels of *MMP-2* were detected in six of the eight MFH tumors as compared to none of the BF cases ($p = 0.022$). Similarly when combining the groups of MFH and AF, which are both characterized by a tendency to recur locally, high mRNA-expression of *MMP-2* was seen in ten of the fifteen cases as compared to none of the six BF cases ($p=0.008$, Table 3). Furthermore the four MFH patients who died from the disease or who developed a local recurrence during followup all had high expression of *EMMPRIN* while this was not the case for any of the four MFH patients who remained disease free during

**Table 3.** *High expression (+++, >70% positive cells) of mRNAs in relation to tumor type*

| Subtype | *EMMPRIN* | p-value* | *MMP-2* | p-value* | *MT1-MMP* | p-value* |
|---|---|---|---|---|---|---|
| MFH | 4/8 (50%) | n.s | 6/8 (75%) | 0.022 | 1/5 (20%) | n.s |
| AF | 3/7 (43%) | n.s | 4/7 (57%) | n.s | 0/6 (0%) | n.s |
| MFH+AF | 7/15 (47%) | n.s | 10/15 (67%) | 0.008 | 1/11 (9%) | n.s |
| BF | 1/6 (17%) | n.s | 0/6 (0%) | n.s | 0/5 (0%) | n.s |
| Total (MFH + AF+BF) | 8/21 (38%) | | 10/21 (48%) | | 1/16 (6%) | |

MFH = malignant fibrous histiocytoma; AF = aggressive fibromatosis; BF = benign fibrous tumor
*P-value is calculated as compared to BF; n.s. = not significant

**Table 4.** *MFH tumors with high expression (+++, >70% positive cells) of EMMPRIN-, MMP-2- or MT1-MMP-mRNA in relation to clinical outcome*

| | DOD or local rec. | NED | p-value |
|---|---|---|---|
| *EMMPRIN* | 4/4 (100%) | 0/4 (0%) | 0.0143 |
| *MMP-2* | 4/4 (100%) | 2/4 (50%) | n.s |
| *MT1-MMP* | 1/2 (50%) | 0/3 (0%) | n.s |

DOD = dead of disease; NED = no evidence of disease; n.s = not significant follow up. This association was also statistically significant (p=0.014, Table 4).

### Discussion

A major characteristic of the malignant phenotype is the ability to invade the surrounding stroma and subsequently to metastasize. These events require degradation of the basement membrane and of the components of the extracellular matrix. Several metalloproteinases and their regulators have previously been shown to play important roles in the invasion process of various tumors.[21] In the present study the mRNA-expression of *EMMPRIN, MMP-2* and *MT1-MMP* were demonstrated in three types of mesenchymal tumors with different biological behaviour, indicating that the matrix degrading system is frequently activated in these tumors. In general, the detection of mRNA expression for a given gene cannot be taken as an evidence of a corresponding protein expression. However, the high expression of *MMP-2* mRNA in MFH and AF is in agreement with previous reports where a strong immunohistochemical reactivity for *MMP-2* was found in the same type of tumors.[22,24] A significant correlation between the expression of *MMP-2*- and *MT1-MMP* mRNA and localisation of the corresponding antibodies as demonstrated by immunohistochemistry has also been demonstrated in head and neck tumors.[23] These findings indirectly indicate that the production of mRNA *de facto* corresponds to a synthesis of the corresponding proteins.

The coexpression of *EMMPRIN*- and *MMP-2* mRNAs in the majority of the tumors suggests that *EMMPRIN* could be of importance for the initiating of *MMP-2* production also in mesenchymal tumors. We found in agreement with previous reports for *MMP-2*, but not for *EMMPRIN*, an mRNA expression in stroma-like and endothelial cells as well as in neoplastic cells which might reflect their common mesenchymal origin.[21] *EMMPRIN* mRNA has previously been demonstrated in both malignant and non-cancerous cells *e.g.* keratocytes. *MMP-2* expression in both benign and malignant cells as has been demonstrated in head and neck carcinoma which may represent one of the mesenchymal characteristics that are acquired during the malignant transformation. These preliminary data with regard to mesenchymal tumors should be interpreted with caution considering the difficulty to distinguish between normal stroma cells such as fibroblasts and atypical cells that are frequently seen outside the tumor pseudo-capsule.

A correlation between MMP production and tumor characteristics such as invasiveness and metastatic capacity has earlier been shown in several reports.[13] In the present study the mRNA-expression of *MMP-2* was significantly more frequent in MFH and AF than in BF. Although both BF and AF lack metastatic potential they frequently expressed *MMP-2* mRNA, indicating that *MMP-2* itself is not an indicator of metastases. However, the limited tumor material in the present study does not allow any conclusions to be drawn regarding the role of *MMP-2* in the process of tumor progression.

All tumors from MFH patients who had a local recurrence or died of the disease, demonstrated high levels of *EMMPRIN* mRNA whereas this was not seen in any of the tumors from patients that remained disease-free. The role of *EMMPRIN* as a prognostic marker in MFH remains to be elucidated. These preliminary findings are in agreement with previous reports and indicate that activation of the extracellular matrix degrading system could be an important component in the aggressive behaviour of MFH tumors.[13]

### Acknowledgement

This study was financially supported by grants from the Swedish Medical Research Council, the Swedish Cancer Foundation, the Torsten and Ragnar Söderberg Foundations and the Cancer Society of Stockholm. The authors would like to thank Roland Perfekt for valuable advises on the statistical analyses.

### References

1 Enzinger FM, Weiss SW. *Malignant fibrohistiocytic tumors*. Mosby, St. Louis, 1995.

2 Weiss SW, Einzinger FM. *Malignant fibrous histiocytoma*. An analysis of 200 cases. *Cancer* 1978; 42:2250–2266.

3 Broders AC, Hargrave R, Meyerding HW. Pathological features of soft tissue fibrosarcoma with special reference to grading of its malignancy. *Surg Gynecol Obstet* 1939; 69:267–280.

4 Angervall L, Kindblom LG, Rydholm A, Sterner B. The diagnosis and prognosis of soft tissue tumors. *Semin Diagn Pathol* 1986; 3:240–258.

5 Le Doussal V, Coindre JM, Leroux A, Hacene K, Terrier P NB, Bonichon F, Collin F, Mandard AM, Contesso G. Prognostic factors for patients with localized primary malignant fibrous histiocytoma: a multicenter study of 216 patients with multivariate analysis. *Cancer* 1996; 77:1823–1830.

6 Pritchard DJ, Reiman HM, Turcotte RE, Ilstrup DM. Malignant fibrous histiocytoma of the soft tissues of the trunk and extremities. *Clin Orthop* 1993; 289:58–65.

7 Rooser B, Willen H, Gustafson P, Alvegard TA, Rydholm A. Malignant fibrous histiocytoma of soft tissue. A population-based epidemiologic and prognostic study of 27 patients. *Cancer* 1991; 67:499–505.

8 Duffy M. Inhibiting tissue invasion and metastasis as targets for cancer therapy. *Biotherapy* 1992; 4:45–52.

9 Chambers AF, Matrisian LM. Changing views of the role of matrix metalloproteinases in metastasis. *J Natl Cancer Inst* 1997; 89:1260–1270.

10 Melchiori A, Albini J, Stetler-Stevenson WG. Inhibition of tumor cell invasion by a highly conserved peptide sequence from the matrix metalloproteinase enzyme prosegment. *Cancer Res* 1992; 52:2353–2356.

11 Tomita T, Iwata K. Matrix metalloproteinases and tissue inhibitors of metalloproteinases in colonic adenomas—adenocarcinomas. *Dis Colon Rectum* 1996; 39:1255–1264.

12 Sato H, Takino T, Okada Y, Cao J, Shingawa A, Yamamoto E, Seiki M, A. Matrix metalloproteinase expressed on the surface of invasive tumour cells. *Nature* 1994; 370:61–65.

13 Kähäri VM, Saarialho-Kere U. Matrix metalloproteinases and their inhibitors in tumour growth and invasion. *Ann Med* 1999; 31:34–45.

14 Miyauchi T, Kanekura T, Yamaoka A, Ozawa M, Miyazawa S, Muramatsu T. Basigin, a new, broadly distributed member of the immunoglobulin superfamily, has strong homology with both the immunoglobulin V domain and the beta-chain of major histocompatibility complex class II antigen. *J Biochem (Tokyo)*. 1990; 107:316–323.

15 Miyauchi T, Masuzawa Y, Muramatsu T. The basigin group of the immunoglobulin superfamily: complete conservation of a segment in and around transmembrane domains of human and mouse basigin and chicken HT7 antigen. *J Biochem (Tokyo)* 1991; 110:770–774.

16 Biswas C, Zhang Y, DeCastro R, Guo H, Nakamura T, Kataoka H, Nabeshima K. The human tumor cell-derived collagenase stimulatory factor (renamed EMMPRIN) is a member of the Immunoglobulin superfamily. *Cancer Res* 1995; 55:434–439.

17 Guo H, Zucker S, Gordon MK, Toole BP, Biswas C. Stimulation of matrix metalloproteinases production by recombinant extracellular matrix metalloproteinase inducer from transfected Chinese Hamster ovary cells. *J Biol Chem* 1997; 272:24–27.

18 Dalberg K, Eriksson E, Enberg U, Kjellman M, Bäckdahl M. Gelatinase A, membrane type 1 matrix metalloproteinase, and extracellular matrix metalloproteinase inducer mRNA expression: correlation with invasive growth of breast cancer. *World J Surg* 2000; 24:334–340.

19 Collier IE, Wilhelm SM, Eisen AZ, Marmer BL, Grant GA, Seltzer JL, Kronberger A, He CS, Bauer EA, Goldberg GI. H-ras oncogene-transformed human bronchial epithelial cells (TBE-1) secrete a single metalloprotease capable of degrading basement membrane collagen. *J Biol Chem* 1988; 263:6579–6587.

20 Zedenius J, Ståhle-Bäckdahl M, Enberg U, Grimelius L, Larsson C, Wallin G, Bäckdahl M. Stromal fibroblasts adjacent to invasive thyroid tumors: expression of gelatinase A but not stromelysin 3 mRNA. *World J Surg* 1996; 20:101–106.

21 Duffy MJ, McCarthy K. Matrix metalloproteinases in cancer: Prognostic markers and targets for therapy (Review). *Int J Oncol* 1998; 12:1343–1348.

22 Soini Y, Salo T, Oikarinen A, Autio-Harmainen H. Expression of 72 Kilodalton and 92 Kilodalton Type IV Collagenase in malignant fibrous histiocytomas and dermatofibromas. *Lab Invest* 1993; 69:305–311.

23 Hurskainen T, Soini Y, Tuuttila A, Höyhtyä M, Oikarinen A, Autio-Harmainen H. Expression of the tissue metalloproteinase inhibitors TIMP-1 and TIMP-2 in malignant fibrous histiocytomas and dermatofibromas as studied by In situ hybridization and immunohostochemistry *Hum Path* 1996; 27:42–49.

24 Imanishi Y, Fujii M, Tokumaru Y, Tomita T, Kanke M, Kanzaki J, Kameyama K, Otani Y, Sato H. Clinical significance of expression of membrane type 1 metalloproteinase and matrix metalloproteinase-2 in human head and neck squamous cell carcinoma. *H Path* 2000; 31:895–904.
